# Heme Oxygenase-1 and Inflammation in Experimental Right Ventricular Failure on Prolonged Overcirculation-Induced Pulmonary Hypertension

**DOI:** 10.1371/journal.pone.0069470

**Published:** 2013-07-25

**Authors:** Asmae Belhaj, Laurence Dewachter, François Kerbaul, Serge Brimioulle, Céline Dewachter, Robert Naeije, Benoît Rondelet

**Affiliations:** 1 Laboratory of Physiology, Faculty of Medicine, Université Libre de Bruxelles, Brussels, Belgium; 2 Service de Chirurgie Cardiovasculaire et Thoracique, Hôpital Mont-Godinne, Université Catholique de Louvain, Yvoir, Belgium; 3 Service de Chirurgie Thoracique, Hôpital Erasme, Université Libre de Bruxelles, Brussels, Belgium; 4 Département d’Anesthésie et Réanimation, Hôpital La Timone, Université de Marseille, Marseille, France; 5 Service des Soins Intensifs, Hôpital Erasme, Université Libre de Bruxelles, Brussels, Belgium; Indiana University, United States of America

## Abstract

Heme oxygenase (HO)-1 is a stress response enzyme which presents with cardiovascular protective and anti-inflammatory properties. Six-month chronic overcirculation-induced pulmonary arterial hypertension (PAH) in piglets has been previously reported as a model of right ventricular (RV) failure related to the RV activation of apoptotic and inflammatory processes. We hypothesized that altered HO-1 signalling could be involved in both pulmonary vascular and RV changes. Fifteen growing piglets were assigned to a sham operation (n = 8) or to an anastomosis of the left innominate artery to the pulmonary arterial trunk (n = 7). Six months later, hemodynamics was evaluated after closure of the shunt. After euthanasia of the animals, pulmonary and myocardial tissue was sampled for pathobiological evaluation. Prolonged shunting was associated with a tendency to decreased pulmonary gene and protein expressions of HO-1, while pulmonary gene expressions of interleukin (IL)-33, IL-19, intercellular adhesion molecule (ICAM)-1 and -2 were increased. Pulmonary expressions of constitutive HO-2 and pro-inflammatory tumor necrosis factor (TNF)-α remained unchanged. Pulmonary vascular resistance (evaluated by pressure/flow plots) was inversely correlated to pulmonary HO-1 protein and IL-19 gene expressions, and correlated to pulmonary ICAM-1 gene expression. Pulmonary arteriolar medial thickness and PVR were inversely correlated to pulmonary IL-19 expression. RV expression of HO-1 was decreased, while RV gene expressions TNF-α and ICAM-2 were increased. There was a correlation between RV ratio of end-systolic to pulmonary arterial elastances and RV HO-1 expression. These results suggest that downregulation of HO-1 is associated to PAH and RV failure.

## Introduction

Pulmonary arterial hypertension (PAH) is a rare and fatal disease characterized by abnormal pulmonary vasoconstriction and pulmonary arteriolar remodeling both leading to a progressive increase in pulmonary vascular resistance (PVR) and eventual right ventricular (RV) failure [1]. Despite recent advances achieved in the management of PAH, the prognosis of PAH patients remains poor, with low quality of life and high mortality rate in the majority of them [2]. This may be related to limited efficacy of targeted therapies in decreasing PVR and pulmonary arteriolar remodeling imposing an increasingly larger load on the RV. The patient outcome is predominantly determined by the response of the RV to the increased afterload [3]–[4]. Little is known about the mechanisms responsible for the development of RV dysfunction on PAH.

Chronic systemic-to-pulmonary shunting in growing piglets has been shown to reproduce in a 3-month period of time typical PAH [5]–[7] and in 6-month typical RV failure [6] features that may require decades of life to develop in patients. In this experimental end-stage PAH model, we previously reported that RV failure was associated with myocardial activation of apoptotic and inflammatory processes [8], like also observed in RV failure on transient pulmonary artery banding in dogs [9]–[10], suggesting common features in the pathobiology of acute and chronic RV failure.

The inducible isoform of heme oxygenase, the HO-1, plays critical roles in regulating inflammatory and cytoprotective processes [11]. HO-1 catalyses the degradation of heme into carbon monoxide, biliverdin and iron [12]. Its activation potentially participates in cellular defense, oxidative stress reduction, inhibition of the activation of inflammation and apoptosis, all due to removal of heme and because of the biological activity of HO-1 products.

CO is an effective pulmonary vasodilator [13], which may act similarly to nitric oxide (NO), activating soluble guanylate cyclase and elevating cGMP production. It inhibits platelet aggregation, reduces leucocyte adhesion, decreases apoptosis and lowers the production of pro-inflammatory cytokines [14]–[15]. Via these properties, HO-1 could be therefore implicated in the pathogenesis of PAH and RV failure, controlling inflammatory phenotype.

In the present study, we took advantage of lung and myocardial tissue stored during previous experiments in pigs with advanced PAH-induced RV failure after 6-month chronic systemic-to-pulmonary shunting to determine the expression of anti-inflammatory and cytoprotective HO-1 and to further explore the activation of inflammatory processes in pulmonary hypertensive disease and RV failure.

## Materials and Methods

The present study was approved by the Institutional Ethics Committee on Animal Welfare from the Faculty of Medicine at the *Université Libre de Bruxelles* (Brussels, Belgium) (Permit Number: 181N) and was done in accordance with the “Guide for the Care and Use of Laboratory Animals” of the United States.

### Protocol - Data Acquisition and Analysis

Fifteen 18±1 days old piglets weighing 5.4±0.3 Kg were assigned to a sham operation (n = 8) or to an anastomosis between the left inominate artery and the pulmonary artery trunk (n = 7), as previously reported [5]–[8]. After 6-month follow-up, the animals were anaesthetized and equipped with catheters and an ultrasonic flow probe placed around the pulmonary artery, as previously described [5]–[8]. Hemodynamic evaluation was performed after shunt closure to avoid flow-sensitive variability. Flow-resistive properties of the pulmonary vessels were further assessed by multipoint mean pulmonary artery pressure (mPpa)/cardiac index (Q) plots obtained by rapid inflation of the inferior vena cava balloon [16]. The ventricular function was estimated by pressure-volume relationships obtained by a ‘single beat method’ used to evaluate RV afterload by pulmonary arterial elastance (Ea) and RV contractility by end-systolic elastance (Ees), and the adequacy of the RV systolic function adaptation to the afterload by the Ees/Ea ratio [17]. At the end of the protocol, the animals were euthanatized with a barbituric overdose. Lung and RV tissue were sampled, immediately snap-frozen in liquid nitrogen and stored at -80°C for biological analysis or after overnight fixation, embedded in paraffin for morphometric evaluations. Lung tissue and RV free wall were collected and stored from 7 sham and 7 shunted pigs during previous experiments [8] and from one sham pig during new experiments.

### Morphometry

Pulmonary arterial morphometry was performed as previously described [5]–[8]. Only the smallest pulmonary resistive arterioles with an external diameter (ED) of <75 µm and a complete muscular coat were measured. Medial thickness (MT) was related to arterial size with the following formula: %MT = (2xMT/ED) × 100 and performed by counting at least 50 pulmonary arteries per lung section from each pig.

### Real-time Quantitative Polymerase Chain Reaction (RTQ-PCR)

Total RNA was extracted from snap-frozen pulmonary and myocardial tissue using the QIAGEN RNeasy**™** Mini kit (QIAGEN, Hilden, Germany), according to the manufacturer’s instructions. Concentration of total RNA was determined by standard spectrophotometric techniques and RNA integrity was assessed by visual inspection of GelRed (Biotium, Hayward, CA)-stained agarose gels. Reverse transcription was performed using random hexamer primers and Superscript™ II Reverse Transcriptase (Invitrogen, Carlsbad, CA, USA), according to the manufacturer’s instructions.

For RTQ-PCR, sense and antisense primers were designed using Primer3 program for porcine heme oxygenase (HO)-1, HO-2, tumor necrosis factor (TNF)-α, intercellular adhesion molecule (ICAM)-1, ICAM-2, vascular cell adhesion protein (VCAM)-1, interleukin (IL)-33, interleukin 1 receptor-like 1 (ST2), IL-19 and signal transducer and activator of transcription (STAT)-3 mRNA sequences (Table 1). To avoid inappropriate amplification of residual genomic DNA, intron-spanning primers were selected when exon sequences were known. For each sample, amplification reaction was performed in triplicate using SYBR-Green PCR Master Mix (Quanta Biosciences, Gaithersburg, MD, USA), specific primers and diluted template cDNA. Result analysis was performed using iCycler System (Biorad Laboratories). Relative quantification was achieved with the comparative 2^−ΔΔCt^ method by the normalization with the housekeeping gene (hypoxanthine phosphoribosyltransferase-1, HPRT1) [18].

**Table 1:** Primers used for RTQ-PCR in porcine lung and myocardial tissue.

Genes	Primer Sequences
HO-1	
Sense	5′-ATGTGAATGCAACCCTGTGA-3′
Antisense	3′-GGAAGCCAGTCAAGAGACCA-5′
HO-2	
Sense	5′-CGCAGCAGTTCAAGCAGTT-3′
Antisense	3′-CCTCCTCCACGATCTTCTCTT-5′
TNF-α	
Sense	5′-TCTGGACTTTGCTGAATCTGG-3′
Antisense	3′-TGAGGGGGTCTGAAGGAGTAA-5′
ICAM-1	
Sense	5′-GCTATCTTGGGCAGTGTTGG-3′
Antisense	3′-AGGCTGGTGTGCTAAGTTTCA-5′
ICAM-2	
Sense	5′-GCCCACTTTTGTGACCGTAG-3′
Antisense	3′-GGTGATGGTGAGGGTTTCA-5′
VCAM-1	
Sense	5′-GAGTTAATCCGGTTGGGACA-3′
Antisense	3′-TTCACAGAACTGCCCTCCTC-5′
IL-33	
Sense	5′-CCGGCAAAGTCTCGATAAAA-3′
Antisense	3′-ATGATAAGGCCAGAGCGAAG-5′
ST2	
Sense	5′-CCTGGAGTTCATTCCCTCTG-3′
Antisense	3′-GGAGATTGTTGGTGCTCCTT-5′
IL-19	
Sense	5′-CGAGGTCTCAGGAGGTGTCT-3′
Antisense	3′-GATGGTGACATTTTGCATGG-5′
STAT3	
Sense	5′-CGCAGAGTTCAAACACCTGA-3′
Antisense	3′-AGCTCCTCGGTCACAATGAG-5′

### Western Blotting

Proteins were extracted from snap-frozen pulmonary and myocardial tissue by homogenization in an appropriate ice-cold lysis buffer, as previously described [19]. After centrifugation, protein concentration was determined using the Bradford protein assay. Protein extracts (50 µg) were resolved on 4–12% NuPage Bis-Tris gels (Invitrogen, Carlsbad, CA, USA) and electro-transferred to nitrocellulose membranes (BioRad, Hercule, CA, USA). After blocking with 5% non-fat milk in 0.1% Tween-TBS (Tris–HCl (pH 8.0); NaCl 150 mM), membranes were incubated with rabbit anti-HO-1 (1∶500; Sigma-Aldrich, St Louis, MO, USA) monoclonal antibody. After incubation with secondary HRP-conjugated anti-mouse antibody (1∶25000; ThermoScientific, Rockford, IL, USA), immunoreactive bands were detected using SuperSignal**™** WestPicoChemiluminescent substrate (ThermoScientific, Rockford, IL, USA) and quantified by laser densitometry (Bio 1D software; Viber Lourmat, Marne La VAllée, France). Relative quantification was systematically performed by normalization with β-actin (Sigma-Aldrich, St Louis, MO, USA) on each blots. Samples from sham and shunted pigs were randomly tested in each Western Blot and one randomly chosen reference sample was deposited on all different blots to allow the comparison and the quantification of all different blots together.

### Statistical Analysis

Values are reported as mean ± standard error of the mean (SEM). Statistical analyses were performed using StatView 5.0 Software. The hemodynamic and biological data of 6-month shunt and 6-month sham groups were compared by performing one-way analysis of variance (one-way ANOVA). When the F ratio of this analysis resulted in a P-value of 0.05 critical value, comparisons were made with a modified two-tailed Student’s t-test [20]. P-values 0.05 were considered statistically significant. Five-point mPpa/Q plots were obtained in all animals, and always best described by a linear approximation, with correlation coefficients higher than 0.95. A linear regression was calculated on each of them by the least squares method Plots of mPAP as a function of Q were compared by t-tests on slopes or on interpolated mPAP at cardiac indexes of 2 and 5 L.min^−1^.m^−2^.

## Results

### Hemodynamic and Morphometric Evaluation

Six-month chronic systemic-to-pulmonary shunting increased mean pulmonary artery pressure (mPpa) (sham (17±1 mmHg) vs shunt (22±2 mmHg), p = 0.04), pulmonary vascular resistance (PVR) (sham (2.6±0.2 mmHg.L^−1^.min.m^−2^) vs shunt (6.4±1 mmHg.L^−1^.min.m^−2^), p = 0.01) and occluded Ppa (Ppao) (sham (7±1 mmHg) vs shunt (11±1 mmHg), p = 0.0004) with no change in mean systemic artery pressure (mPsa). As already partially reported elsewhere [8], the mPpa/Q relationships were shifted to higher pressures with an increase in slope ^hello^sham (3.4±0.4 mmHg.L^−1^.min.m^−2^) vs shunt (7.2±0.6 mmHg.L^−1^.min.m^−2^), p = 0.0003] (8). There was a marked decrease in Ees/Ea ratio (sham (1.51±0.06) vs shunt (0.68±0.07), p = 0.000001) [8], indicating a RV-arterial uncoupling in the 6-month shunted compared with the 6-month sham-operated pigs. Cardiac index (Q) (sham (3.5±0.3 L.min^−1^.m^−2^) vs shunt (1.9±0.1 L.min^−1^.m^−2^), p = 0.0002) [8] and stroke volume decreased (sham (42±5 mL.Beat^−1^) vs shunt (27±5 mL.Beat^−1^), p = 0.003) and right atrial pressure (Pra) tended to increase (sham (6.1±0.3 mmHg) vs shunt (7.1±0.5 mmHg), p = 0.09).

**Figure 1:**
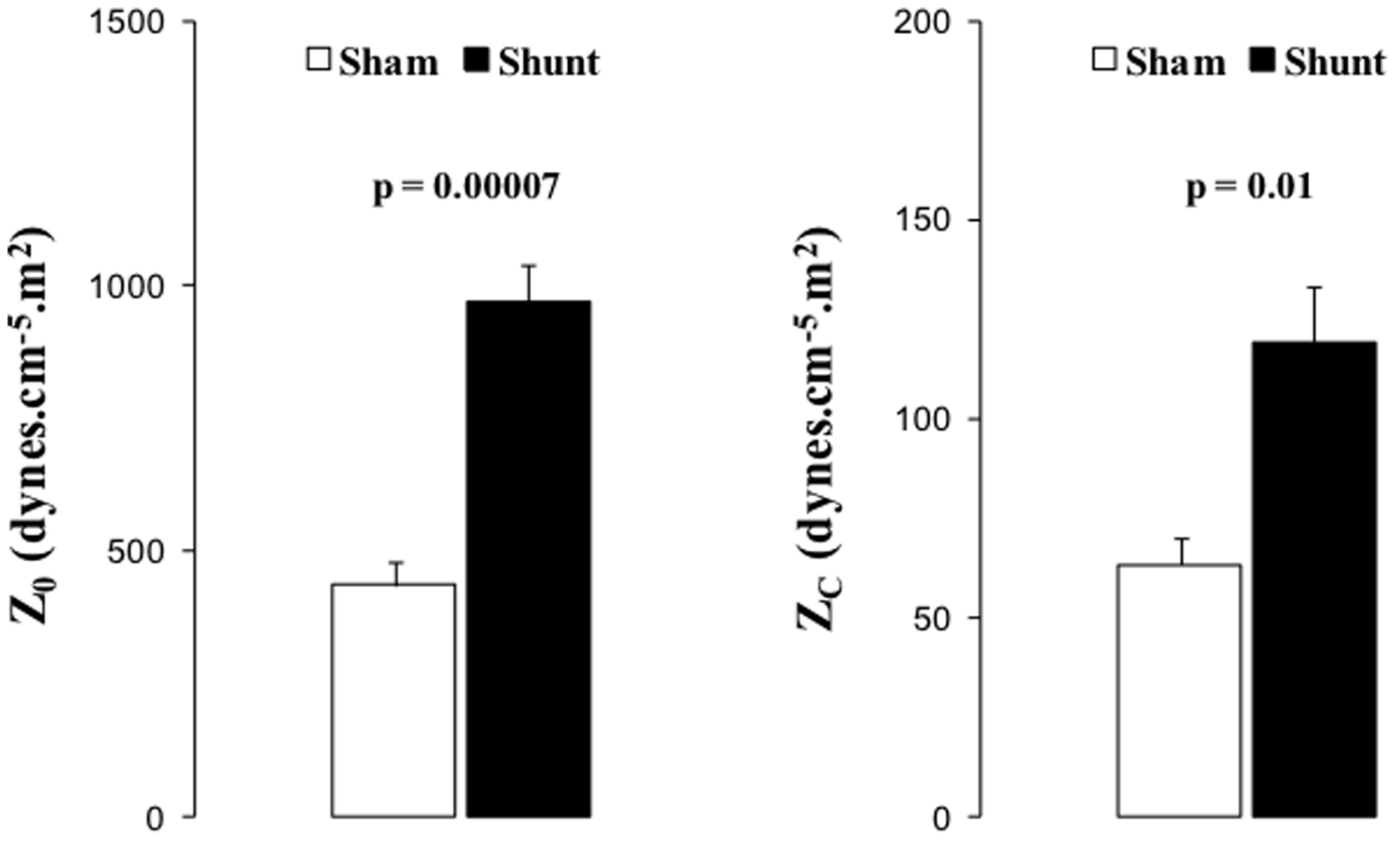
Pulmonary hemodynamics. Zo and Zc. Black squares or columns: shunt, empty circles and empty columns: sham piglets. Values expressed as mean±SEM. P values indicate significances on comparisons of means of Z0 and Zc.

Six-month aorta-pulmonary shunting was associated with increased Z_0_ and Z_C_ impedance (Figure 1).

**Figure 2:**
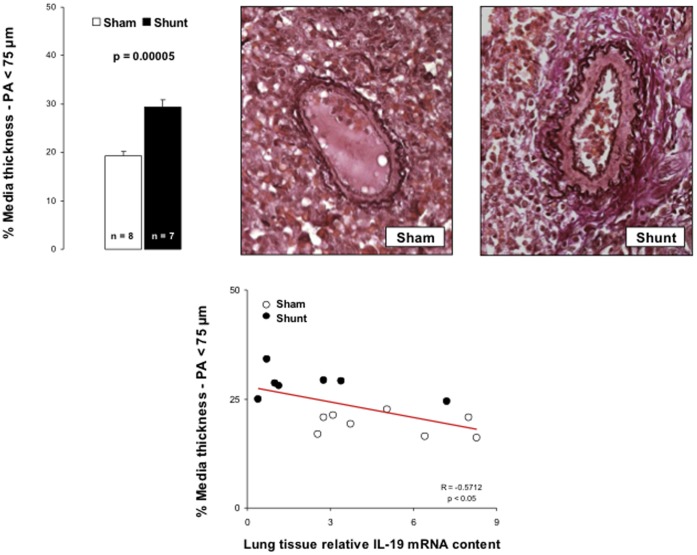
Morphometry on smallest pulmonary arterioles (<75 micrometers) obtained in Sham and Shunt piglets and labeled by the method of von Gieson-orcein. At least 50 resistive arterioles have been measured per animal. The medial thickness (MT) has been reported in vessel diameter following the formula %MT = [(2× MT)/ED]×100 where ED is the external diameter of arterioles measured. MT in pulmonary arteries under 75 micrometers correlated to lung tissue relative IL-19 mRNA content. Values expressed as mean±SEM.

After 6-month systemic-to-pulmonary shunting, pulmonary arterial thickness increased, mostly in the smallest pulmonary arteries (<75 µm) (Figure 2).

### Pulmonary Hypertensive Disease - Pulmonary Expressions of Heme Oxygenases and Cell Adhesion Molecules

**Figure 3:**
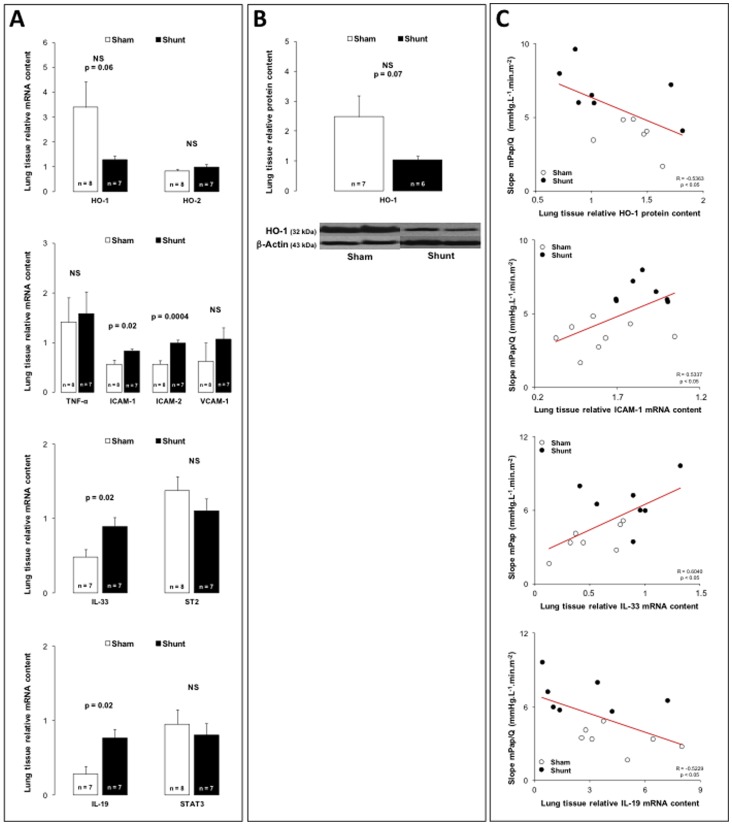
Panel A: Relative lung tissue mRNA content for the heme-oxygenase(HO)-1 and -2 tumor necrosis factor(TNF)-α, intercellular adhesion molecule(ICAM)-1 and -2, vascular cell adhesion molecule(VCAM)-1, interleukin(IL)-33, interleukin 1 receptor-like 1 (ST2), interleukin(IL)-19 and signal transducer and activator of transcription(STAT)-3 in 6-month sham (white bars) and 6-month shunt (black bars) piglets. Panel B: Relative lung tissue protein content for the heme-oxygenase(HO)-1 in 6-month sham (white bars) and 6-month shunt (black bars) piglets. Panel C: Correlation between slope of mPpa/Q relationships and lung protein content for heme-oxygenase(HO)-1, lung mRNA content for intercellular adhesion molecule(ICAM)-1, interleukin(IL)-33 and interleukin(IL)-19. Values expressed as mean±SEM.

Systemic-to-pulmonary shunting was associated with a non significant but strong tendency to the decreased gene (p = 0.06) and protein (p = 0.07) expressions of inducible HO-1 isoenzyme in lung tissue (Figure 3A and 3B), while pulmonary gene expression of constitutive HO-2 isoenzyme did not change (Figure 3A). As illustrated in Figure 3A, pulmonary gene expressions of cell surface glycoproteins mediating inflammatory cell adhesion, such as ICAM-1 and ICAM-2 increased, while pulmonary expression of VCAM-1 did not change.

### Pulmonary Hypertensive Disease - Pulmonary Expressions of Tumor Necrosis Factor(TNF)- α, Interleukins (IL )-33 and -19

As illustrated in Figure 3A, pulmonary gene expression of pro-inflammatory TNF-α did not change after 6-month systemic-to-pulmonary shunting.

In the 6-month shunted group, pulmonary gene expression of IL-33, a pro-inflammatory cytokine belonging to the IL-1 cytokine family, increased, while pulmonary expression of ST2, a decoy receptor which negatively regulates IL-1/IL-33 signaling pathway, did not change (Figure 3A).

Six-month systemic-to-pulmonary shunting increased pulmonary gene expression of anti-inflammatory IL-19, a cytokine belonging to the IL-10 cytokine family, while pulmonary expression of STAT3, the transcription factor downstream activated, remained unchanged (Figure 3A).

### Right Ventricular Failure – RV Expressions of Heme Oxygenases and Inflammatory Mediators

**Figure 4:**
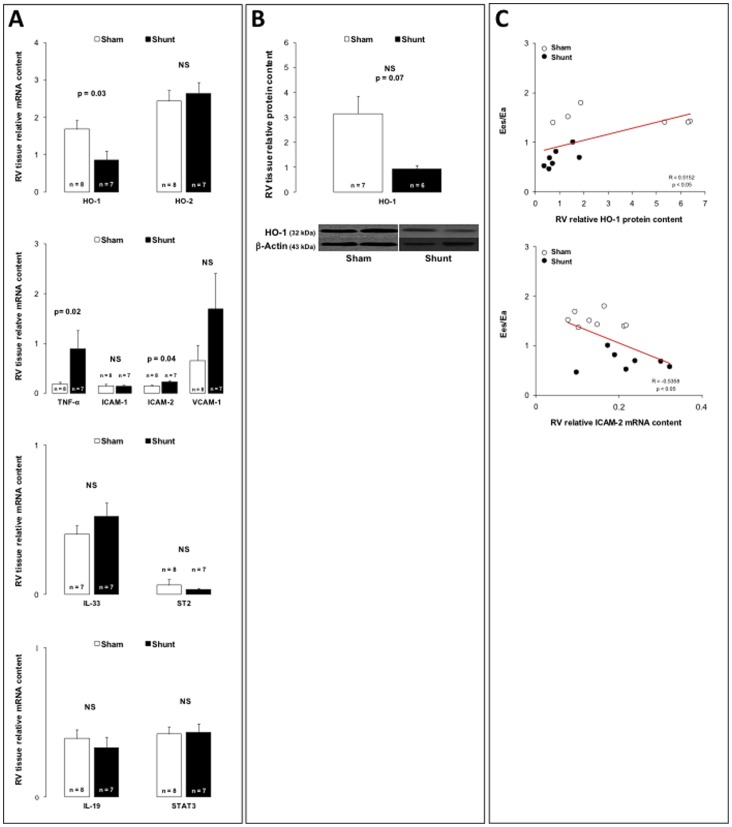
Panel A: Relative right ventricular (RV) tissue mRNA content for the heme-oxygenase(HO)-1 and -2, tumor necrosis factor(TNF)-α, intercellular adhesion molecule(ICAM)-1 and -2, vascular cell adhesion molecule(VCAM)-1, interleukin(IL)-33, interleukin 1 receptor-like 1 (ST2), interleukin(IL)-19 and signal transducer and activator of transcription(STAT)-3 in 6-month sham (white bars) and 6-month shunt (black bars) piglets. Relative right ventricular (RV) tissue protein content for the heme-oxygenase(HO)-1 in 6-month sham (white bars) and 6-month shunt (black bars) piglets. Correlation between Ees/Ea ratio and right ventricular (RV) protein content for heme-oxygenase (HO)-1 and right ventricular (RV) mRNA content for intercellular adhesion molecule (ICAM)-2. Values expressed as mean±SEM.

As illustrated in figure 4, systemic-to-pulmonary shunting decreased the RV relative gene expression of inducible HO-1 isoenzyme (Figure 4A), while RV protein expression tended to decrease, but not significantly (p = 0.07) (Figure 4B). RV gene expression of constitutive HO-2 expression remained unchanged (Figure 4A).

RV failure was associated with increased RV gene expressions of TNF-α and ICAM-2, while RV expressions of ICAM-1, VCAM-1, IL-33, ST2, IL-19 and STAT3 remained unchanged (Figures 4A).

### Correlations

To assess whether there might have been a link between the inflammatory phenotype and the development of PAH, we looked for correlations between typical PAH features, such as the PVR (obtained as the slope of multipoint mPpa/Q plots) or the pulmonary arteriolar remodeling and the pulmonary expressions of HO-1 and biological determinants implicated in inflammatory processes. PVR was inversely correlated to pulmonary expressions of ICAM-1 (Figure 3C), ICAM-2 and pro-inflammatory IL-33 (Figure 3C) and inversely correlated to pulmonary expressions of cytoprotective and anti-inflammatory HO-1 (Figure 3C) and anti-inflammatory IL-19 (Figure 3C). There was a negative correlation between the percentage of medial thickness of pulmonary arterioles <75 µm and the pulmonary gene expression of IL-19 (Figure 2).

To understand better the link between inflammatory phenotype and RV adaptation to the increased afterload, we also looked for correlations between Ees/Ea ratio and different inflammatory determinants. There was a positive correlation between RV Ees/Ea ratio and RV protein expression of cytoprotective and anti-inflammatory HO-1 (Figure 4C) and a negative correlation between with RV ICAM-2 gene expression (Figure 4C).

## Discussion

The present results suggest that decreased HO-1 expression and local activation of inflammatory processes are associated to both pulmonary vascular and RV remodeling in pulmonary hypertension on six-month chronic systemic-to-pulmonary shunting in growing piglets.

We previously reported on RV failure induced by 6 months of aorta-pulmonary shunting in growing piglets [8]. The present study further explored pulmonary hemodynamics and associated biological changes in these animals. Pulmonary artery impedance measured at 0Hz (Z_0_) and characteristic impedance (Z_c_) were increased, which is in keeping with previous report on 3-month piglets [21]. Right ventricular afterload and hydrolic load is best described by a pulmonary arterial impedance (PVZ) spectrum, which integrates pulmonary vascular resistance (PVR), elastance, and wave reflection [16], [21]. Increases in Z_0_, or total PVR, calculated as Ppa/Q, may be related with the changes in medial thickness in distal and resistive arterioles whereas increases in characteristic impedance (Z_c_), ratio between the inertance and compliance of the proximal pulmonary arterial tree [21], could be interpreted as a decrease of pulmonary artery compliance related with structural and/or functional modifications in proximal pulmonary artery. PVR was defined by multipoint mPAP-flow plots. This approach is superior to single point PVR calculations as the slope of mPAP-flow may be less than predicted by the PVR equation because of recruitment, distension or both [22].

Inflammation has been shown to play roles in human and experimental pulmonary hypertension (PH) and is increasingly recognized as a major pathogenic component of the pulmonary vascular remodeling in pulmonary arterial hypertension (PAH) [23]. Moreover, disturbances in homeostatic processes regulating neurohumoral activation, oxidative stress, inflammation and apoptosis have also been suggested to participate to RV failure in PH [24]. We have recently reported that RV failure on prolonged systemic-to-pulmonary shunting in growing piglets [8] and on transient pulmonary artery banding in dogs [9], [10] are associated with increased activation of apoptosis and inflammation, including overexpressions of pro-inflammatory cytokines. This reinforces the notion that inflammation seems to play crucial roles in the pathogenesis of PAH and RV failure.

In the present study, pulmonary expressions of ICAM-1 and ICAM-2 were increased after 6-month systemic-to-pulmonary shunting, while VCAM-1 expression did not change. The ICAM glycoproteins which are normally weakly expressed by endothelial cells, have been shown to be upregulated after injury and responsible for the orchestration of the recruitment and the attachment of inflammatory cells to the endothelium. This represents a marker of endothelial activation [25], acting as a prelude to the migration of inflammatory cells into other vascular layers and probably contributing to the vascular remodeling. Pulmonary overexpression of ICAM-1 has been observed in rats with monocrotaline injection [26]- and chronic hypoxia exposure [27]–[28]-induced PH. Moreover, increased flow pulsatility has been shown to induce endothelial expression of inflammatory genes, including ICAM-1 [29], suggesting that prolonged shunt-induced overcirculation could contribute to the development of an inflammatory phenotype in the lungs of the present experimental model. In accordance with the present results showing a tight relation between pulmonary ICAM-1 expression and the PVR, the severity of the pulmonary hypertensive disease has been tightly correlated to serum level of soluble ICAM-1 in patients with congenital heart disease and PH [2].

In the present experimental model of PAH, IL-33 was overexpressed in the lungs, while expression of its ST2 receptor remained unchanged. Recently described member of the IL-1 cytokine family, IL-33 is a strong inducer of T helper 2 (Th2) immune responses [30] and contributes to the early events in endothelial activation, promoting endothelial expression of adhesion molecules (e.a. ICAM-1 and VCAM-1) and pro-inflammatory chemokines (e.a. monocyte chemoattractant protein-1) [31]. IL-33 could therefore contribute to the endothelial activation and subsequent pulmonary arterial remodeling in PAH. Normally released by necrotic cells as an “alarming factor” alerting the immune system to tissue damage or stress, mechanical strain has also been shown to induce the secretion of IL-33 in fibroblasts in the absence of cellular necrosis [32]. Via its binding to the ST2 receptor, IL-33 also strongly induces Th2 cytokine production (e.a. IL-4, -13 and -19) from these cells and can promote the pathogenesis of Th2-related disease, such as pulmonary arterial remodeling [33].

Six-month systemic-to-pulmonary shunting increased pulmonary expression of IL-19, while STAT3 expression did not change. This could be seen as a Th2-related cytokine production. In vascular smooth muscle cells, IL-19 rapidly evokes the activation and the translocation of STAT3 transcription factor [34] which has been recently incriminated in the development of idiopathic PAH [35] and experimental monocrotaline injection-induced PH [36]. IL-19 also induces the expression of the potent inflammatory modulator HO-1 and decreases the production of reactive oxygen species in human vascular smooth muscle cells [17]. IL-19 has been shown to decrease dose-dependently the proliferation of vascular smooth muscle cells [15], [34], [37]–[38], whereas, in endothelial cell, HO-1 induction increases cell cycle progression [39]. Increased pulmonary IL-19 expression could be therefore partly responsible for the non-aggravation of the pulmonary vascular remodeling in the 6-month compared to the 3-month shunted pigs [5], [8].

In the present study, we found a trend to the decreased expression of HO-1 in the lungs after 6-month systemic-to-pulmonary shunting. Overexpression of HO-1 has been shown to prevent the development of hypoxia-induced PH, associated with reduced pulmonary inflammation (characterized by increased macrophage activity and IL-10 expression [40])and vascular remodeling [41]–[42]. HO-1 mediates *in vivo* the protective effects in monocrotaline-induced PH and *in vitro* the anti-proliferative effects in smooth muscle cells of rapamycin [43]. In contrast, pulmonary HO-1 expression has been shown to be increased in monocrotaline- and monocrotaline/pneumectomy-induced PH [44].

Here, we also showed decreased gene expression of HO-1 in the failing RV. These results suggest a similar tendency to changes in HO-1 in the lungs and the failing RV in the present experimental model of advanced PAH, probably through divergent HO-1 enzymatic products [45]. Indeed, biliverdin has been shown to prevent RV dysfunction, but did not reduce the pulmonary artery remodeling, while inhaled CO reduced the pulmonary vascular remodeling, but did not have any effect on the RV (45). In mice exposed to chronic hypoxia, administration of mesenchymal cells overexpressing HO-1 has been shown to reduce the RV hypertrophy, to stabilize the infarction zones and to decrease the RV systolic pressure to normal values [46]. The RV HO-1 expression has been shown to be increased and decreased respectively in experimental models of RV pressure overload [47] and RV failure [48]. In patients with end-stage congenital heart disease, RV levels of HO-1 were increased with variable magnitude [49]. This suggests that HO-1 expression seems to depend on the stress-induced cardiomyocyte damage and the evolution of RV failure.

In the present study, the decreased HO-1 expression was related to the activated inflammatory phenotype in the RV, as assessed by increased RV ICAM-2 expression and previously shown increased RV IL-1α, IL-1β and TNF-α expressions [8]. Moreover, the RV IL-33 expression did not change. IL-33 is mainly produced by cardiac fibroblasts in response to mechanical strain. In the left ventricle, IL-33 has been shown to prevent cardiomyocyte hypertrophy though its soluble ST2 receptor [50]. After chronic exposure to pressure overload induced by transverse aortic constriction, transgenic ST2-deficient mice developed more ventricular hypertrophy, cavity dilations and fibrosis and had only a limited survival rate. Moreover, IL-33 administration reduced ventricular hypertrophy and fibrosis and improved the survival rate in the control mice but not in ST2-deficient mice [50]. Mechanically activated IL-33/ST2 signalling could be, therefore, responsible for a cardioprotective paracrine system regulated by cardiac fibroblasts. In the present experimental model of RV failure, myocardial gene expressions of IL-33 and ST2 remained unchanged in the 6-month shunted and sham pigs. This observation suggests that the cardioprotective mechanism depending on the activation of the IL-33/ST2 signalling pathway fails to be activated in the failing RV and probably implicated in the pathogenesis of RV failure.

In conclusion, the present study shows that PAH and RV failure are associated with downregulation of HO-1 signaling and local activation of inflammatory processes.
